# Soliris to Stop Immune-Mediated Death in COVID-19 (SOLID-C19)—A Compassionate-Use Study of Terminal Complement Blockade in Critically Ill Patients with COVID-19-Related Adult Respiratory Distress Syndrome

**DOI:** 10.3390/v13122429

**Published:** 2021-12-03

**Authors:** Thomas C. Pitts

**Affiliations:** Hudson Medical, 281 Broadway, 2nd Fl., New York, NY 10007, USA; drpitts@hudsonmedical.com

**Keywords:** eculizumab, soliris, coronavirus, COVID-19, complement, ARDS, acute respiratory distress syndrome

## Abstract

Eculizumab, a terminal complement (C5)-inhibiting monoclonal antibody, was administered in five mechanically ventilated patients in life-threatening condition due to COVID-19-related acute respiratory distress syndrome (ARDS) between 23 March 2020 and 3 April 2020. Their clinical progress was monitored. The primary endpoint was mortality. One patient was excluded while two passed away. The remaining two patients survived. At the time of this study, the mortality rate in mechanically ventilated COVID-19 patients suffering from ARDS receiving the standard of care as their therapeutic regimen was reportedly as high as 97%. This pilot study demonstrates a 50% mortality rate in patients receiving eculizumab therapy.

## 1. Introduction

**Figure 1:**
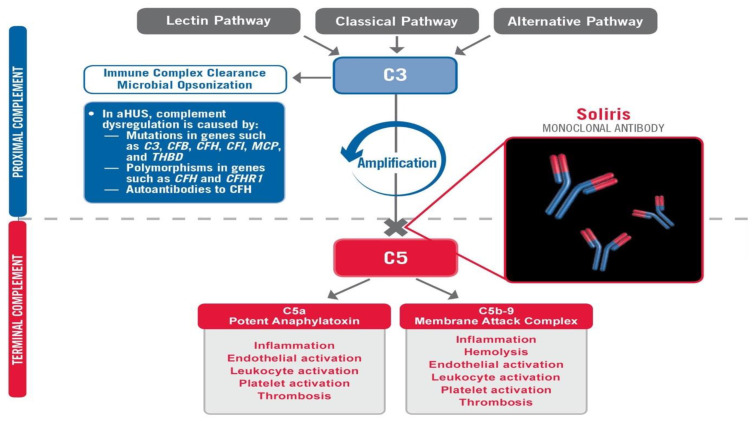
Eculizumab anti-C5 mechanism of action.

COVID-19 has spread rapidly throughout the world, causing widespread injury and death. While the virus is the provocateur, it is often the patient’s own disproportionate immune response which deals the most devastating (and often fatal) damage. The Membrane Attack Complex (MAC) is a primary mediator of this catastrophic, immune-mediated, end-organ damage, and is formed by the various components of the terminal complement [1]. The MAC is ordered to form and attack when C1q, a surveillance component of the complement system, surveys the antigen and calls upon its destruction, even if the involvement of the MAC causes devastating destruction to the end organ within which the virus exists [2]. This mechanism has been shown to be a mechanism of injury in other subclasses of coronaviruses [3]. In the SOLID-C19 compassionate-use study, Soliris (Eculizumab) was used to modulate the activity of the terminal complement by preventing the formation of the MAC via C5 inhibition Figure 1. By modulating this portion of the immune response, mortality can potentially be halted while the patient has time to recover from the virus with supportive medical care. The proximal complement is left intact. The MAC’s primary role is to combat certain encapsulated bacteria; thus, its inhibition should stop immune-mediated end-organ destruction and thrombosis while not hindering viral fighting components of the immune system. This small pilot study aimed to achieve effective targeting of the terminal complement to halt catastrophic MAC-mediated lung damage and acute respiratory distress syndrome (ARDS) in intubated COVID-19 patients in life-threatening condition.

## 2. Materials and Methods

To be included in the study, all patients had to be age 18 or older (Table 1) with a confirmed COVID-19 infection requiring mechanical ventilation due to ARDS. Patients were excluded if they were suffering from an active Neisseria infection, as this is a contraindication to eculizumab use. Patients received Neisseria meningococcal prophylactic antibiotic therapy while taking Soliris. Patients were also excluded if they were already enrolled in another experimental immunosuppressive therapy trial. FDA emergency investigational new drug (eIND) applications were submitted, and FDA emergency use authorizations were obtained for each patient. The primary endpoint was mortality. Initially, 11 patients were enrolled across four sites, each with an approved FDA emergency use authorization. Unfortunately, six patients were never able to receive the medication as they passed away before the medication, which was shipped emergently and directly from the manufacturer, arrived (Table 2). Six of the patients enrolled were males and one patient was below the age of forty (Table 3). 

## 3. Results

The SOLID-C19 trial’s first patient was a 44-year-old female patient A1 who was taking hydroxychloroquine and mycophenolate mofetil chronically for lupus. The patient called her rheumatologist on 12 March 2020, concerned about a possible lupus flare complaining of intermittent fever, cough, and generalized myalgias including chest discomfort when breathing. The patient’s rheumatologist stopped her mycophenolate mofetil and started her on 40 mg po daily prednisone with a 12-day taper at that time. On 16 March 2020 the patient went to her primary care physician because these symptoms were becoming more frequent and bothersome, and she now reported dyspnea when laying supine. The patient even noticed some crackles when she listened to her own lungs. Rhonchi in the left upper lung were noted on physical exam. Rapid flu and urinalysis were normal. Chest X-ray showed left midlung disease. It was speculated that this could be a developing pneumonia and so doxycycline was started. She was sent to the emergency department for COVID-19 testing and went home after the test, reportedly in no acute distress. She was told to quarantine for 14 days as she was exposed to COVID-19-positive patients where she worked.

The patient subsequently reported to the emergency department on 18 March 2020 with worsened dyspnea as well as lightheadedness. Electrocardiogram was normal. Chest X-ray showed mild to moderate bibasilar opacities. Her oxygen saturation was 88% during this evaluation on room air and she had a fever of 38.8 °C. CT PE study was obtained and remonstrated the bibasilar opacities and was negative for pulmonary embolism. She was admitted to the hospital. That night she had worsening dyspnea and remained febrile at 39 °C. She was intubated and transferred to the intensive care unit. At the time of intubation her blood pressure was 84/45 and her respiratory rate was 43/min. Repeat chest X-ray after intubation showed significant progression of the bilateral lung disease consistent with acute respiratory distress syndrome (ARDS). C-reactive protein at the time of intubation was 33.7. Flu and RSV were negative. The patient was started on piperacillin/tazobactam and norepinephrine by the medical critical care team.

While intubated, the patient continued to experience desaturation of her oxygen. Her PaO2 was 71 while intubated and she had a generalized seizure. Her ventilator settings were adjusted and her SPO2 rose to 95%. A chest X-ray was again obtained which remonstrated findings consistent with severe ARDS with superimposed bilateral pulmonary edema. Furosemide 40 mg IV was given at that time. The patient was moved to a rotaprone bed and turned to the prone position as she continued to experience intermittent desaturations while supine, despite mechanical ventilation.

Certriaxone 2 gm every 12 h was ordered by the infectious disease team and piperacillin/tazobactam was discontinued. Azithromycin was also added. She remained on hydroxychloroquine and her steroids were stopped. Neurology was consulted who ruled out CNS disease and attributed the seizure to hypoxia. The patient’s husband and three children were brought into the quarantine area to say goodbye to the patient as they were informed that she would likely pass away. 

The consulting neurologist, Dr. Eleina Mikhaylov, notified the author to see if the patient would qualify for the Soliris to Stop Immune-Mediated Death in COVID-19 Infected Patients compassionate-use study (SOLID-C19-NCT04288713 clinicaltrials.gov, accessed 28 February 2021). The author and Dr. Mikhaylov obtained consent from the patient’s husband to enroll the patient in the trial after local IRB and FDA eIND approvals were obtained. Eculizumab was sent to the facility emergently and was given at 22:30 on 23 March 2020.

The patient’s Murray score Table 4 was 13/16 prior to the first dose. She was given eculizumab 900 mg IV over 35 min for each dose. Her Rocephin served as her meningococcal prophylaxis. The seroB and quadrivalent meningococcal vaccines were also administered by the infectious disease team. 

**Figure 2:**
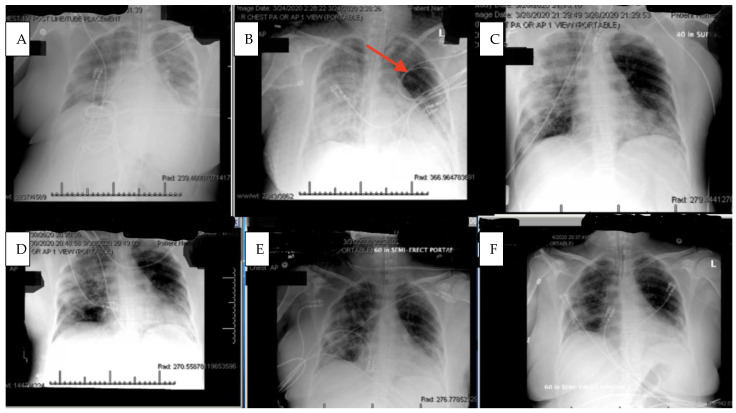
Chest X rays prior to and during eculizumab therapy. (**A**): Chest X ray taken 22 h prior to first dose of eculizumab deonstrates bilateral extensive pulmonary infiltration consistent with Covid-19 related Adult Respiratory Distress Syndrome. (**B**): four hours after the first eculizumab dose (red arrow denotes area of improved aeration). (**C**): Following the second dose of eculizumab. (**D**): Following the third dose of eculizumab. (**E**): Following the fourth dose of eculizumab. (**F**): Extubation.

Within 4 h after the first dose of eculizumab, her chest X-ray improved, Figure 2, and her Murray score improved from 13 to 10. She had a daily improvement in Murray score except on 27 March 2020 when her endotracheal tube moved out of place while she was prone and had to be readjusted, in which case her Murray score rose slightly but still remained lower than her pretrial baseline. Another 900 mg IV eculizumab dose was given 4 days after the first dose and, again, her chest X-ray improved. Her hydroxychloroquine was stopped by the infectious disease team on 28 March 2020 due to a possible contribution to a normocytic anemia. Norepinephrine was discontinued on 29 March 2020 as her blood pressure stabilized. The patient was able to be adequately oxygenated in the supine position and her rotabed was discontinued on 31 March 2020. On 2 April 2020, the patient’s third dose of eculizumab 900 mg IV was given and her Murray score improved from 8/16 on 1 April 2020 to 6/16 recorded seven hours after the third dose of eculizumab was given on 2 April 2020 Figure 3. On 3 April 2020 the medical critical care team started to wean the patient’s sedation (propofol, fentanyl, and midazolam). The patient passed a spontaneous breathing trial and followed appendicular commands on 4 April 2020. Mechanical ventilation was continued to allow the sedation to fully wear off in preparation for extubation. The patient’s Murray score had decreased to 3/16, she was able to oxygenate in the supine position, remained afebrile, and did not require presser medications. She was successfully extubated on 5 April 2020, Figure 3. She had amnesia and some bradyphrenia but was otherwise neurologically normal and even “face-timed” on the phone with family a few hours subsequent to extubation. Arm swelling and pain was noted and an upper extremity DVT was discovered. The patient completed a course of inpatient rehabilitation and was discharged home. She completes all ADLs and IADLs on her own.

After this first case, 11 other critically ill and mechanically ventilated COVID-19 patients received emergency use approval from the FDA for Eculizumab (Table 5). Six of these patients either passed away prior to the medication arrival or had significantly worsened by the time the medication arrived and were not deemed likely to survive. Five patients were dosed in total with Eculizumab. One patient was clinically improving but suffered a self-extubation resulting in their death and was excluded from the study as the death was deemed to be non-trial related (Table 6). In total, two patients survived and were extubated, and two patients passed away (Table 7). Adverse events included amnesia and one case of upper extremity venous thrombosis (Table 8). The results demonstrate an overall mortality in critically ill and mechanically ventilated COVID-19 patients who received eculizumab of 50%, compared to estimates as high as 97% for patients receiving the standard of care at the time of this study. 

## 4. Discussion

Identifying the highest value approach for a patient is not always clear. While many efforts have been made to treat the virus itself in COVID-19 patients, this case highlights the vital role of immunomodulation to halt the catastrophic complement mediated lung damage that occurs COVID-19-related ARDS [3]. Successful extubation was facilitated by modulation of this immune-mediated damage and allowing the body’s normal antiviral mechanisms to clear the virus (as with most coronaviruses that we regularly encounter) with supportive medical care. The implication is that the virus may not be as high value a target as the disproportionate immune response that it can provoke. Complement activation can lead to prothrombotic and proinflammatory states Figure 1. Not surprisingly, patients have suffered from DVT, stroke, PE, and AMI in addition to ARDS in the setting of COVID-19-induced complement activation.

COVID-19 is scrutinized by C1q [4], likely provoked by the expression of IgG1 and IgG3 (as can be seen in anti-acetylcholine receptor antibody-mediated Myasthenia Gravis and various other complement-activating autoimmune diseases which may share common HLA haplotype mutations) to initiate the overwhelming, disproportionate, and often lethal complement-mediated immune response, which directs the MAC against the virus and injures the lungs and other end organs such as the kidney and nervous system in the process. It additionally contributes to proinflammatory cytokine activation via C5a activation while promoting a prothrombotic state leading to DVT, PE, AMI, or stroke in many [3].

The study’s first case, patient A1, is especially important because the patient also had lupus and a history of positive SS-B and ANA antibodies in her blood. It is well-described that patients with autoimmune diseases may have exacerbations of their conditions in the setting of illness and immune activation (i.e., Myasthenia Gravis crisis with an infection or surgery), and it is plausible that this patient had to not only contend with the immunological onslaught provoked by the virus but also from her chronic autoimmune disease (especially as her mycophenolate mofetil and subsequently her hydroxychloroquine were stopped). It is also well-described that immune-mediated damage can continue long after the inciting infectious pathogen has been cleared from the body (i.e., viral or bacterial provoked Guillain–Barre syndrome leading to CIDP). In fact, while the patient tested positive for COVID-19 initially, her COVID-19 test drawn on the day of extubation was negative, which shows that the immune-mediated damage can continue after the virus becomes undetectable in the blood. It is important to note that we immunomodulate Guillian–Barre patients in this situation rather than trying to treat them with antiviral medications, as their misguided and overwhelming immune response is the primary cause of end-organ damage which is likely the case in COVID-19-mediated ARDS. 

It is also critical to point out that the patient was intubated and almost died while on hydroxychloroquine chronically and did not clinically improve with the concomitant administration of azithromycin. It was not until eculizumab was delivered that the patient began to improve, Figure 2. The patient also continued to improve despite hydroxychloroquine being stopped by the infectious disease consultant due to a concern for anemia. Her upper extremity DVT may have been provoked from indwelling lines and immobility over several weeks on mechanical ventilation. Complement inhibition typically works against complement induced prothrombotic states. COVID-19 is known to provoke DVTs, pulmonary emboli, strokes, and a general tendency towards a prothrombotic state in many patients. 

In a disease state for which certain data suggests as many as 97% of intubated patients with COVID-19 related ARDS die [5], inhibition of the membrane attack complex with eculizumab was effective in saving 50% of the studied patients and helped to facilitate extubation and ultimately discharge them from the hospital. While this is compassionate-use data in a small group of patients, the high mortality rate in the general COVID-19-related ARDS population can feasibly serve as a “natural control” to some degree. In any event, this is an extremely promising therapeutic approach and merits further direct clinical implementation and study. This is especially true because, in the process of halting the catastrophic immune response, eculizumab does not inhibit viral fighting mechanisms. Eculizumab safety is substantiated in numerous studies as well as almost two decades of case studies for its four other FDA indications (AchR + generalized myasthenia gravis [6], atypical hemolytic uremic syndrome [7], paroxysmal nocturnal hemoglobinuria [8], and anti-AQP4 neuromyelitis optica [9]). There are also several case studies citing the safety of eculizumab in pregnant patients [10]. It is vital that we act promptly during this troubling time and facilitate the investigation of promising and possibly lifesaving therapies which have been proven safe for almost two decades [11]. This is exceptionally true given the fact that ICUs and hospitals are, in many places, becoming overwhelmed and often in short supply of invasive ventilators. This study was closed, and all data were submitted to the FDA. An FDA expanded-access study was approved following the submission of the SOLID C-19 pilot study data.

## 5. Conclusions

Terminal complement inhibition preventing formation of the membrane attack complex and its associated catastrophic end-organ damage [3] is a promising and relatively safe approach [11] in COVID-19-related ARDS. This was demonstrated in this small but promising pilot study, especially in our first patient who suffered from concomitant lupus who was intubated and did not improve despite being on hydroxychloroquine chronically or with the addition of azithromycin. After the administration of eculizumab, the objective data show a clear and sustained clinical improvement and preservation of life compared with the general standard of care available at the time of this study. Our first case may also reinforce recommendations for patients suffering with autoimmune diseases to obtain their COVID-19 vaccine despite a potential risk of vaccine-induced provocation of these illnesses via typical antibody forming and immunological mechanisms needed to form a protective antibody, as, if they were to contract COVID-19, this autoreactive immune response may prove to be much more prolonged and possibly catastrophic. Although complement activation has been associated with the pathophysiology of ARDS caused by various underlying diseases, clinical data on the role of complement activation in the development of SARS-CoV-2-associated ARDS are scarce [12] and this study should serve to encourage further investigation.

## Figures and Tables

**Figure 3:**
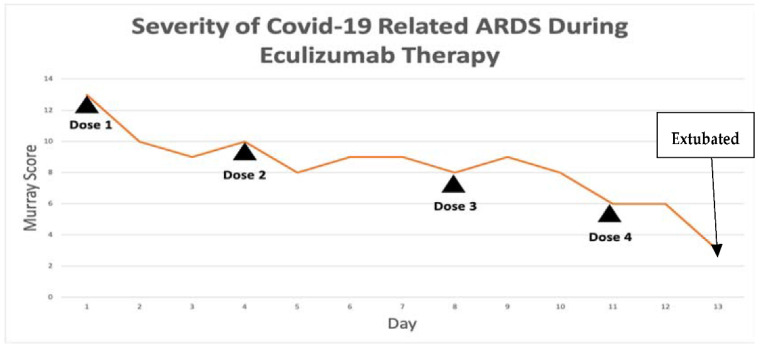
Severity of COVID-19-related ARDS over time during Eculizumab therapy for patient A1.

**Table 1:** Inclusion and Exclusion Criteria.

Inclusion Criteria	Exclusion Criteria
18 years old or older	Active Neisseria Infection
Confirmed COVID-19	Current enrollment in another immunosuppresant study
ARDS	
ICU patient	

**Table 2:** Subject Enrollment by Site.

Site	Total Enrolled	First Enrollment Date	Last Enrollment Date
Premier Health–Upper Valley Medical Center (Dayton/Troy, OH)	1	23 March 2020	23 March 2020
Montefiore Hospital–Bronx, NY	8	30 March 2020	3 April 2020
Detroit Medical Center, Detroit, MI	1	2 April 2020	2 April 2020
Southside Hospital-Bayshore, NY	1	1 April 2020	1 April 2020
Total US sites	11	23 March 2020	3 April 2020
Total non-US sites	0	N/A	N/A
All Sites	11	23 March 2020	3 April 2020

**Table 3:** Subject Demographics.

Patient Age (Years)	Female	Male	Total
18–21	0	0	0
22–29	0	0	0
30–39	0	1	1
40–49	1	2	3
50–59	1	1	2
60–69	0	2	2
70–79	2	1	3
>80	0	0	0

**Table 4:** Murray Score.

	Murray Score				
Variable	0	1	2	3	4
Chest X ray (Quadrant)	Normal	1	2	3	4
Positive End Expiratory Pressure (cm H_2_O)	<5	6–8	9–11	12–14	>15
Compliance (mL/cm H_2_O)	>80	60–79	40–59	20–39	<19
PaO2/FiO2 (On 100% Oxygen) in mm Hg	>300	225–299	175–224	100–174	<100

**Table 5:** Status of Enrolled Participants.

	Overall
Total planned for enrollment	11
Total enrollment at completion	11
Total Who Received Medication	5
On Treatment Currently	0
Completed Treatment	2
Total Terminated Study	9
Termination Associated with an Adverse Event	0
Termination Due to Subject Death	3 *
Screen Failures	6 *
Other (define based on your situation)	0

* Deaths—Two patients died of very advanced illness including ARDS/respiratory failure, and renal failure related to COVID-19. The third patient died from accidental self extubation complications and was improving clinically prior to this. * Screening Failures—Screening failures all had approved eINDs but their clinical situation worsened prior to arrival of the medication from the manufacturer to the point that the medication was withheld as survival was not likely.

**Table 6:** Patients Who Received Treatment.

Patient ID	FDA Emergency Use Authorization Approved?	Endpoint-Mortality	Summary of Clinical Observations
A1	Yes	Alive	Daily improvement after eculizumab started. Extubated. Discharged
B2	Yes	Deceased	ARDS, intubated, renal failure, and high inflammatory markers.
C3	Yes	Alive	Daily improvement after eculizumab started. Extubated. Discharged
D4	Yes	Deceased	ARDS, intubated, renal failure, and high inflammatory markers.
E5	Yes	Deceased	Self-extubated—Non-trial related

**Table 7:** Adverse Events: Frequent and/or Serious.

Body System	N	Incidence
Infections and infestations	0	0
Injury, poisoning and procedural complications	0	0
Nervous system disorders *	1	1
Respiratory, thoracic and mediastinal disorders	0	0
Blood and lymphatic system disorders	0	0
Musculoskeletal and connective tissue disorders	0	0
Gastrointestinal disorders	0	0
General disorders and administration site conditions	0	0
Hepatobiliary disorders	0	0
Skin and subcutaneous tissue disorders	0	0
Eye disorders	0	0
Ear and labyrinth disorders	0	0
Psychiatric disorders	0	0
Vascular disorders *	1	1
Immune system disorders	0	0
Metabolism and nutrition disorders	0	0
Renal and urinary disorders	0	0
Reproductive system and breast disorders	0	0
Surgical and medical procedures	0	0

Neurological *—Amnesia. Vascular *—Upper extremity deep vein thrombosis.

**Table 8:** Adverse Event by Subject.

Subject ID	Adverse Event	Expected?	Likely Study Related?
E5	Death	No	No—Self-extubation. Was clinically improving.
B2	Death	Yes	No—Died of pulmonary, cardiac, and renal failure due to COVID-19
D4	Death	Yes	No—Died of pulmonary, cardiac, and renal failure due to COVID-19
